# Retraction Note: Therapeutic effect of nanoliposomal PCSK9 vaccine in a mouse model of atherosclerosis

**DOI:** 10.1186/s12916-023-02884-w

**Published:** 2023-05-04

**Authors:** Amir Abbas Momtazi-Borojeni, Mahmoud Reza Jaafari, Ali Badiee, Maciej Banach, Amirhossein Sahebkar

**Affiliations:** 1 Nanotechnology Research Center, Bu-Ali Research Institute, Mashhad University of Medical Sciences, Mashhad, Iran; 2 Department of Medical Biotechnology, Student Research Committee, Faculty of Medicine, Mashhad University of Medical Sciences, Mashhad, Iran; 3 Nanotechnology Research Center, Pharmaceutical Technology Institute, Mashhad University of Medical Sciences, Mashhad, Iran; 4 Biotechnology Research Center, Pharmaceutical Technology Institute, Mashhad University of Medical Sciences, Mashhad, Iran; 5 Department of Pharmaceutical Nanotechnology, School of Pharmacy, Mashhad University of Medical Sciences, Mashhad, Iran; 6 Department of Hypertension, WAM University Hospital in Lodz, Medical University of Lodz, Zeromskiego 113, Lodz, Poland; 7 Polish Mother’s Memorial Hospital Research Institute (PMMHRI), Lodz, Poland; 8 Neurogenic Inflammation Research Center, Mashhad University of Medical Sciences, Mashhad, Iran; 9 School of Pharmacy, Mashhad University of Medical Sciences, Mashhad, Iran

**Retraction Note: BMC Med 17, 223 (2019)**

**https://doi.org/10.1186/s12916-019-1457-8**

The Editor has retracted this article. After publication, concerns were raised regarding the images presented in Fig. 7. Specifically:

The left PHA image in Fig. 7a appears highly similar to the middle PHA image in Fig. 7b (rotated);

All the Medium images in Fig. 7a and b appear highly similar; the left and right images in Fig. 7b appear to be missing some features compared with the other images.

The authors have been able to provide the quantified data, but stated that the original images are no longer available. The Editor therefore no longer has confidence in the presented data.

None of the authors agrees to this retraction.

